# Somatic mutations are present in all members of the *AKT* family in endometrial carcinoma

**DOI:** 10.1038/sj.bjc.6605301

**Published:** 2009-09-08

**Authors:** A Dutt, H B Salvesen, H Greulich, W R Sellers, R Beroukhim, M Meyerson

**Affiliations:** 1 Department of Medical Oncology, Dana-Farber Cancer Institute, 44 Binney Street, Boston, MA 02115, USA; 2 Cancer Program, The Broad Institute of MIT and Harvard, 7 Cambridge Center, Cambridge, MA 02142, USA; 3 Department of Clinical Medicine, The University of Bergen, Bergen 5020, Norway; 4 Department of Obstetrics and Gynecology, Haukeland University Hospital, Bergen N-5021, Norway; 5 Department of Medicine, Brigham and Women's Hospital, 75 Francis Street, Boston, MA 02115, USA; 6 Novartis Institutes for Biomedical Research, 250 Massachusetts Avenue, Cambridge, MA 02139, USA; 7 Department of Cancer Biology, Dana-Farber Cancer Institute, Boston, MA 02115, USA; 8 Department of Medicine, Harvard Medical School, Boston, MA 02115, USA; 9 Center for Cancer Genome Discovery, Dana-Farber Cancer Institute, 44 Binney Street, Boston, MA 02115, USA; 10 Department of Pathology, Harvard Medical School, Boston, MA 02115, USA

**Sir,**

The activating E17K mutations recently discovered in the pleckstrin homology domain of *AKT1* in about 2% of endometrial cancer patients (Shoji *et al*, 2009) suggest a new mechanism for PI3 kinase pathway activation in these patients, as previously described in breast, colorectal and ovarian cancers (Carpten *et al*, 2007). Additional mechanisms of PI3 kinase pathway activation in endometrial cancer include the somatic mutation of *PTEN* and *PIK3CA* (Kong *et al*, 1997; Oda *et al*, 2005), amplification and overexpression of *PIK3CA* (Miyake *et al*, 2008; Salvesen *et al*, 2009), and decreased expression of *PTEN* (Kanamori *et al*, 2001; Kappes *et al*, 2001).

We found an additional four mutations in AKT family members (Table 1). Two of these (mutations in the catalytic domain of *AKT2* (D399N) and the regulatory domain of *AKT3* (E438D)) were previously reported in a sequencing screen of 123 genes in 41 primary endometrial cancers (Dutt *et al*, 2008). Manual reinspection of these data in light of the report on activating *AKT1* mutations in endometrial cancer (Shoji *et al*, 2009) revealed an additional mutation, *AKT1* E17K, identical to the one reported by Shoji *et al* (2009), and a novel mutation in the catalytic domain of *AKT2* (R368C) in two additional samples. We validated these mutations as somatic by mass spectrometric genotyping of the tumour and matched normal DNA, after an independent PCR amplification. We also found a novel candidate mutation in the pleckstrin homology domain of *AKT2* (D32H), which we could not validate because of insufficient DNA. All these mutations occurred in cancers of the endometrioid subtype that had no signs of metastasis either at primary treatment or during follow-up. Taken together, we find that 5 out of 41 endometrial cancers have mutations in AKT family members for a 12% rate.

Confirmation that these novel mutations activate the PI3 kinase pathway awaits their functional characterisation. Notably, all the AKT family member mutations found in our data occur at residues conserved across multiple species (see Supplementary Figure 1). However, three of these five mutations were identified in samples harbouring mutations of *PTEN*, one of which also had amplification of and a mutation in *PIK3CA* (Table 1); the *AKT1* E17K mutation is not associated with either *PTEN* or *PIK3CA* genomic alteration. It is therefore possible that these AKT family mutations have different functional effects from mutations of *PTEN* and *PIK3CA*. Given the importance of the PI3 kinase pathway in endometrial cancer oncogenesis (Salvesen *et al*, 2009), and the emerging therapeutic options for PI3 kinase inhibition (Garcia-Echeverria and Sellers, 2008), the functional effects of all these AKT family mutations should be investigated in appropriate model systems of endometrial cancer.

## Figures and Tables

**Table 1:** AKT family mutations found in endometrial cancer

**Gene**	**Sample ID**	**Mutation**	**Domain**	**PIK3CA amplification** a	**Other mutations**
AKT1	436T	E17K	Pleckstrin homology	No	KRAS (G13D)
AKT2	288T	D399N	Regulatory C-terminal	No	PTEN (D24Y, F341Y, R130Q)
AKT2	426T	R368C	Catalytic kinase	No	CTNNB1 (S37Y)
AKT2	141T	D32H^b^	Pleckstrin homology	No	PTEN (R130Q)
AKT3	192T	E438D	Regulatory C-terminal	Yes	PTEN (R130Q), PIK3CA (R88Q)

a Determined by segmentation analysis of normalised signal intensities from 100K single-nucleotide polymorphism arrays as previously reported (Salvesen *et al*, 2009).

b Candidate mutation not validated by mass spectrometric genotyping.
