# Supplementary material for: Somatic mutations are present in all members of the AKT family in endometrial carcinoma
Source: Br J Cancer. 2009 Sep 8;101(7):1218–9. doi: 10.1038/sj.bjc.6605301 (PMC2768084; doi:10.1038/sj.bjc.6605301)
Supplement: Supplementary Figure 1 [file 6605301x1.pdf]

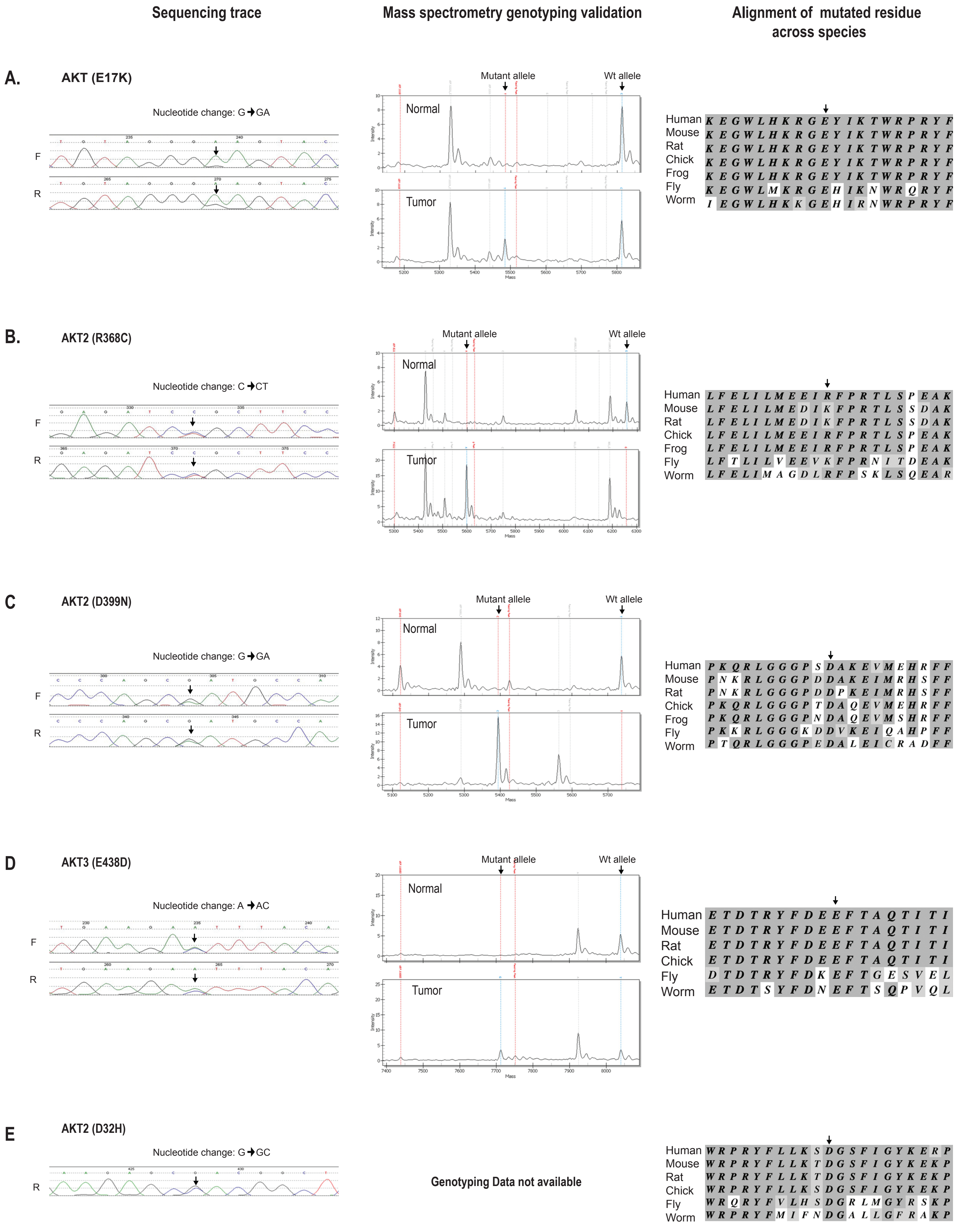
Sequencing trace
Mass spectrometry genotyping validation
Alignment of mutated residue across species
A.
AKT (E17K)
Nucleotide change: G➔GA
F
R
Mutant allele
Wt allele
Normal
Tumor
Human KEGWLHKRGEYIKTWRPRYF
Mouse KEGWLHKRGEYIKTWRPRYF
Rat KEGWLHKRGEYIKTWRPRYF
Chick KEGWLHKRGEYIKTWRPRYF
Frog KEGWLHKRGEYIKTWRPRYF
Fly KEGWLMKRGEHIKNWRQRYF
Worm IEGWLHKKGEHIRNWRPRYF
B.
AKT2 (R368C)
Nucleotide change: C➔CT
F
R
Mutant allele
Wt allele
Normal
Tumor
Human LFELILMEEIRFPRTLSPEAK
Mouse LFELILMEDIKFPRTLSSDAK
Rat LFELILMEDIKFPRTLSSDAK
Chick LFELILMEEIRFPRTLSPEAK
Frog LFELILMEEIRFPRTLSPEAK
Fly LFTLILVEEVKFPRNITDEAK
Worm LFELIMAGDLRFPSKLSQEAR
C
AKT2 (D399N)
Nucleotide change: G➔GA
F
R
Mutant allele
Wt allele
Normal
Tumor
Human PKQRLGGGPSDAKEVMEHRFF
Mouse PNKRLGGGPDDAKEIMRHSFF
Rat PNKRLGGGPDDPKEIMRHSFF
Chick PKQRLGGGPTDAQEVMEHRFF
Frog PKQRLGGGPNDAQEVMSHRFF
Fly PKKRLGGGKDDVKEIQAHPFF
Worm PTQRLGGGPEDALEICRADFF
D
AKT3 (E438D)
Nucleotide change: A➔AC
F
R
Mutant allele
Wt allele
Normal
Tumor
Human ETDTRYFDEEFTAQTITI
Mouse ETDTRYFDEEFTAQTITI
Rat ETDTRYFDEEFTAQTITI
Chick ETDTRYFDEEFTAQTITI
Fly DTDTRYFDKEFTGESVEL
Worm ETDTSYFDNEFTSQPVQL
E
AKT2 (D32H)
Nucleotide change: G➔GC
R
Genotyping Data not available
Human WRPRYFLLKSDGSFIGYKERP
Mouse WRPRYFLLKTDGSFIGYKEKP
Rat WRPRYFLLKTDGSFIGYKEKP
Chick WRPRYFLLKSDGSFIGYKEKP
Fly WRQRYFVLHSDGRLMGYRSKP
Worm WRPRYFMIFNDGALLGFRAKP
Intensity
Mass
