# Supplementary material for: Somatic mutations are present in all members of the AKT family in endometrial carcinoma
Source: Br J Cancer. 2009 Sep 8;101(7):1218–9. doi: 10.1038/sj.bjc.6605301 (PMC2768084; doi:10.1038/sj.bjc.6605301)
Supplement: Supplementary Figure Legend [file 6605301x2.doc]

Legend to Supplementary Fig 1.

Summary of the five somatic mutations found in AKT family members in endometrial cancer. For each mutation, the reverse complements of the forward (F) and reverse (R) sequencing reads are displayed as generated by Mutation Surveyor in the left panel. For the AKT2 D32H mutation, the forward read was noisy and is therefore not shown. In the middle panel, Sequenom genotyping spectra are shown for tumor (bottom) and matched normal (top) sample. Expected locations of the wild-type and mutant allele peaks are indicated by arrows. The right panel displays alignment of Human AKT1 (AAL55732), relative to Mouse AKT1 (NP_033782), Rat AKT1 (NP_150233), Chick AKT1 (NP_990386), Frog AKT1 (NP_001083878), Fly AKT (NP_732113), Worm AKT (NP_001023645); Human AKT2 (NP_001617), relative to Mouse AKT2 (NP_035915), Rat AKT2 (NP_113763), Chick AKT2 (AAD54413), Frog AKT2 (NP_001080091); and Human AKT3 (AAH72892), relative to Mouse AKT3 (NP_035915), Rat AKT3 (NP_113763), Chick AKT3 (XP_419544). Residues identical to the human sequence are indicated in dark grey and similar residues are highlighted in light grey. The mutated residue is indicated with an arrow.
